# Assessment of Fungal and Contamination of Ochratoxin A and Patulin in Foods Susceptible to Contamination in the Yangzhou Market, China

**DOI:** 10.3390/foods13193205

**Published:** 2024-10-09

**Authors:** Qinghua Gong, Zihan Zhang, Peiwen Huang, Bo Wang, Xiangfeng Zheng

**Affiliations:** College of Food Science and Engineering, Yangzhou University, No. 196 West Huayang Road, Yangzhou 225009, China; 18669984808@163.com (Q.G.); wb@yzu.edu.cn (B.W.)

**Keywords:** fungi, mycotoxin, food safety, HPLC

## Abstract

The conducive conditions of warm and humid climates can facilitate mold proliferation and subsequent mycotoxin production during food processing and distribution, thereby posing a potential risk to consumer health. However, there exists a significant lack of research regarding the diversity of molds and the presence of ochratoxin A (OTA) and patulin (PAT) in food products available in the Yangzhou market. This study was conducted to assess OTA contamination levels and fungal presence in 57 cereal-based food samples, as well as PAT contamination levels and fungal presence in 50 types of foods, including apples, hawthorn berries, pears, and their derivatives. Ochratoxin A (OTA) was detected in 17 out of 57 cereal-based food samples, with concentrations ranging from 0.93 to 32.69 μg/kg. The contamination rate was determined to be 31.48%, and no samples exceeded the established regulatory limits. Furthermore, seven apple products were identified as contaminated with patulin (PAT), exhibiting concentrations between 26.85 and 192.78 μg/kg. Additionally, three food samples derived from hawthorn showed PAT contamination levels ranging from 29.83 to 88.56 μg/kg. Through purification on potato dextrose agar (PDA) medium, observation of colony morphology, and analysis of internal transcribed spacer (ITS) sequences, a total of 35 fungal strains belonging to 13 genera were identified in cereal-based foods. The predominant genera in cereals included *Talaromyces*, *Fusarium*, *Aspergillus*, and *Penicillium*. Additionally, twelve fungal strains from five genera (*Penicillium*, *Cladosporium*, *Aureobasidium*, *Curvularia*, and *Alternaria*) were isolated and identified in fruits and their derivatives. The findings indicate that OTA and PAT toxins are one of the important risk factors that threaten consumer health. Furthermore, the contamination of some other toxigenic strains is also a matter of substantial concern, with potential implications for consumer health.

## 1. Introduction

Ochratoxin A (OTA) and patulin (PAT) are secondary metabolites produced by the *Aspergillus* and *Penicillium* genera [1]. These compounds have been recognized as contaminants in a diverse array of food sources, thereby significantly undermining the safety of these products. They are recognized as some of the most critical mycotoxins. Extensive research has elucidated the diverse toxicological impacts of these compounds on various organs, particularly the kidneys and liver. OTA has been associated with potential carcinogenic, teratogenic, and mutagenic effects [2], resulting in its classification as a Group 2B human carcinogen by the International Agency for Research on Cancer (IARC) and the World Health Organization (WHO) [3]. Consequently, the European Commission has enacted regulations to monitor ochratoxin A (OTA) levels in imported foods, establishing maximum permissible limits of 5.0 μg/kg for raw cereals, 3.0 μg/kg for processed cereals, and 10.0 μg/kg for raisins [4]. Moreover, in 2004, the European Union revised regulations stipulating that all baby foods and diet foods intended for special medical use in infants must not contain more than 0.5 μg/kg of OTA [5]. PAT, a highly toxic secondary polyketide metabolite, has raised concerns due to its complex toxicity, leading to increased scrutiny from governmental agencies and consumers about its presence in food. Consequently, various regions have set regulations to limit OTA levels in food products. The Codex Alimentarius Commission, along with the regulatory authorities of the United States and China, have established a maximum permissible limit of 50 μg/kg for PAT in apple juice and apple-derived products [6,7]. In contrast, the European Union has established more stringent regulations regarding PAT levels in apple products. Specifically, the maximum allowable concentration is set at 50 μg/kg for fruit juices and apple beverages, and 25 μg/kg for solid apple products. Furthermore, for liquid or solid apple products intended for infants and young children, the permissible PAT limit is restricted to 10 μg/kg [8].

Cereal products are notably impacted by Ochratoxin A (OTA) contamination, initially identified in corn during the 1960s and later detected in various grains such as barley, wheat, rye, and rice [9,10,11]. Subsequent research has confirmed the presence of OTA in additional food items, including beans, grapes, raisins, wine, nuts, beer, cocoa, and spices. Considering the global annual production of cereal crops surpasses 2 billion tons, these crops are especially susceptible to OTA contamination. This issue becomes increasingly critical with the rising production of cereals [12]. Cereals are a significant source of OTA contamination globally, accounting for about 50% of daily human intake through grain products [13]. Pittet et al. found that 25–40% of cereals worldwide are contaminated with mycotoxins, occurring during cultivation and storage [14]. Additionally, Peter et al. detected OTA in 18 of 29 heated grain samples from Saskatchewan farms, with concentrations between 0.03 and 27 ppm. Furthermore, multiple reports have documented the occurrence of PAT in a variety of commodities, including fruits, cocoa, spices, and coffee [11]. Ji et al. (2017) identified 42 PAT-positive samples out of 137 apple products in China, with contamination levels ranging from 10 to 276.9 μg/kg [15]. PAT contamination is prevalent in international markets. Mahato et al. (2021) found significant PAT levels in apples and apple products in both developed countries like the United States, Spain, Portugal, and Belgium, and developing countries such as South Africa, Argentina, and Turkey. Notably, Argentina, South Africa, Spain, and Portugal have reported PAT in baby food [16]. In addition, studies show that cereals and apples are major sources of OTA and PAT in human diets, posing significant health risks, especially to infants [17,18]. The WHO advises that daily intake of these toxins should not exceed 0.4 μg/kg of body weight, highlighting the need to monitor their levels in common foods.

Recent studies have shown that fungi from the *Aspergillus* and *Penicillium* genera can biosynthesize OTA and PAT [19,20]. Specifically, *Aspergillus* species like *A. ochraceus*, *A. carbonarius*, *A. niger*, and *A. westerdijkiae*, as well as *Penicillium* species *P. verrucosum* and *P. nordicum*, are known OTA producers [21,22]. These strains primarily affect cereals and related products. Aspergillus species, including *A. clavatus* [23], *A. giganteus* [24], and *A. longivesica* [25], as well as *Penicillium* species such as *P. expansum* [26], *P. carneum* [27], *P. clavigerum* [28], *P. concentricum* [29], and *P. coprobium*, have been identified as producers of PAT. These strains produce mycotoxins when contaminating food raw materials, leading to toxin accumulation in both raw and processed foods [30,31]. Additionally, during distribution, environmental adaptation can enhance the growth and toxin production of heat-resistant toxigenic bacterial spores in contaminated food, raising contamination risks. Contamination by OTA or PAT-producing fungi is the main source of these toxins in food. Thus, monitoring mold or toxigenic bacteria is crucial for evaluating OTA or PAT risks in food products.

**Figure 1:**
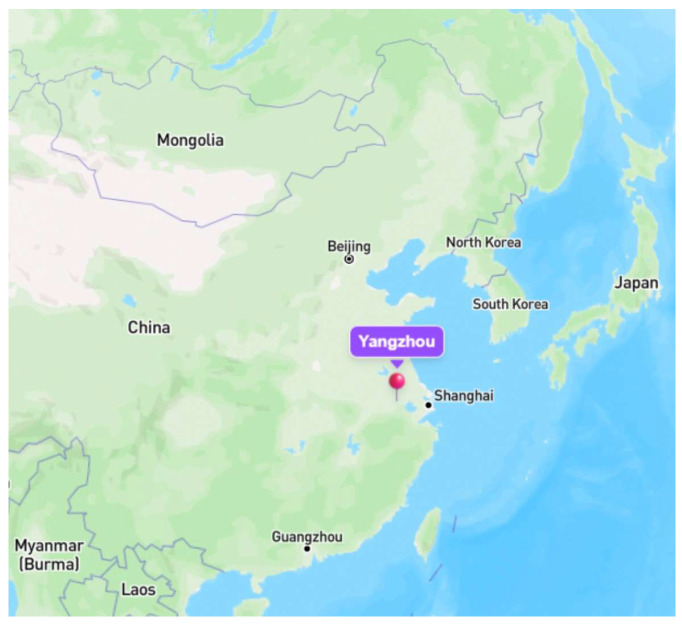
Geographical location of Yangzhou, China.

Yangzhou City, located in the central part of Jiangsu Province at the confluence of the Yangtze River and the Beijing-Hangzhou Grand Canal (Figure 1), lies within the transitional zone between a subtropical monsoon humid climate and a temperate monsoon climate. The hot and humid conditions prevalent in this region foster an optimal environment for fungal proliferation and mycotoxin production. However, there exists a significant lack of research regarding the diversity of molds and the presence of ochratoxin A in cereal products available on the market. Furthermore, there is a limited investigation into the diversity of molds and the contamination of patulin (PAT) in apple and hawthorn products that comply with Chinese food safety standards. This study examined fungal diversity, total fungal count, and ochratoxin A levels in 54 cereal products and related items available in the Yangzhou market. Additionally, it evaluated fungal diversity, total fungal count, and PAT contamination across 43 varieties of foods, including apples, hawthorns, pears, and their derivatives. The findings from this research offer critical insights for governmental market regulation and informed consumer choices.

## 2. Materials and Methods

### 2.1. Chemicals, Reagents, and Standards

Acetonitrile and methanol, both of high-performance liquid chromatography (HPLC) grade, were procured from Tedia Company (Fairfield, CT, USA). OTA and PAT standards were obtained from Pribolab Biological Technical Company (Qingdao, China). Standard OTA solutions were prepared by dissolving the compound in methanol to create a 10 mg/L stock solution. Working standard solutions were subsequently prepared by diluting the stock solution with a mobile phase consisting of acetonitrile and 1% acetic acid in a 6:4 volume/volume (*v*/*v*) ratio. These working standard solutions covered a concentration range from 0 to 100 μg/L, with calibration points at 0, 1, 5, 10, 20, 50, and 100 μg/L. Standard PAT solutions were prepared using a 1 g/L stock solution in methanol. Working standard solutions were subsequently prepared by diluting the stock solution with a mobile phase consisting of methanol and double-distilled water (ddH_2_O) in a 1:9 volume/volume ratio (*v*/*v*). The concentration of the working standard solutions ranged from 0 to 100 μg/L, with specific calibration points at 0.1 μg/L, 1 μg/L, 5 μg/L, 10 μg/L, 20 μg/L, and 100 μg/L. For the phosphate buffer preparation, 8.0 g of sodium chloride, 1.2 g of disodium hydrogen phosphate, 0.2 g of potassium dihydrogen phosphate, and 0.2 g of potassium chloride were weighed and dissolved in approximately 990 mL of water. The pH was adjusted to 7.0 using concentrated hydrochloric acid, and the solution was then diluted with water to a final volume of 1 L. All solutions were filtered using a 0.22 μm membrane (WondaDisc NY organic filter, SHIMADZU-GL Sciences, Shanghai, China) prior to analysis via HPLC. Subsequently, a standard curve was established by correlating the content of OTA or PAT with their respective peak areas.

### 2.2. Sampling Collection and Preparation

A total of 149 samples, comprising 57 varieties of cereal foods, 23 varieties of nut foods, 16 varieties of coffee, 3 varieties of wine, 13 varieties of fruits, and 37 varieties of fruit products (*n* = 3), were randomly collected from supermarkets and farmers’ markets in Yangzhou, China. The collected samples were placed in plastic bags, sealed, labeled, and subsequently transported to the Food Microbiology Analysis Laboratory at Yangzhou University. The samples were stored at room temperature until further analysis. Detailed information regarding the samples is provided in Table 1.

### 2.3. Extraction of OTA from the Samples

Approximately 100 g of each solid sample was powdered using an ultrafine grinder. The resulting powder was sifted through a sifter with a 16-mesh aperture. The filtered sample was then transferred into a polypropylene tube and stored at −20 °C until analysis. For the liquid samples, OTA was extracted directly. Precisely 5 g of each sample was weighed into a shaker bottle, followed by the addition of 20 mL of a 60% acetonitrile solution. The mixture was subjected to ultrasonication at 100 W for 30 min and subsequently centrifuged at 2504× *g* for 10 min. Four milliliters of the supernatant were collected and mixed with 26 mL of phosphate buffer. This mixture was then centrifuged again at 2504× *g* for 10 min. The supernatant was collected, and the residue was discarded. The supernatant was subsequently filtered using a SelectCore OTA Immunoaffinity Cartridge for the purification of OTA in accordance with the manufacturer’s instructions.

### 2.4. Determination of OTA by Using HPLC

The detection of OTA content was performed using High-Performance Liquid Chromatography (HPLC) following the method described by Zheng et al. (2023), with minor modifications [32]. The sample was injected into a ZORBAX SB-C18 HPLC column (4.6 × 50 mm, 5 μm). The analysis was conducted using a Shimadzu LC-20A HPLC system equipped with the aforementioned column. The mobile phase consisted of acetonitrile and 1% acetic acid in a 6:4 (*v*/*v*) ratio, with a flow rate of 1 mL/min. OTA detection was achieved using a fluorescence detector set at an excitation wavelength (λex) of 333 nm and an emission wavelength (λem) of 460 nm, following the injection of 20 μL of the sample.

### 2.5. Extraction of PAT from the Samples

PAT was extracted from the sample following the protocol outlined by Zheng et al. (2023), with minor modifications [33]. Liquid samples (e.g., apple juice, hawthorn juice) were introduced into a homogenizer (TTL-260, Beijing TongTaiLian Technology Co., Ltd., Beijing, China) operating at a rotor speed of 15,000× *g*. Solid samples (e.g., hawthorn chips, fruit peel) were pulverized using a high-speed grinder and subsequently mixed thoroughly. High-viscosity samples, such as fruit peel, were freeze-dried using liquid nitrogen and then immediately ground with a high-speed grinder. Semi-fluid samples, including apple puree, apple jam, and fruit juice with grain, were homogenized using a tissue masher. For patulin extraction, ten micrograms of each homogenized sample was combined with a triploid volume of ethyl acetate and subjected to vigorous shaking for 5 min. The mixture was then allowed to stand at room temperature for 10 min, after which the upper layer was transferred to a separatory funnel. This extraction procedure was repeated twice. The upper layers from each extraction were pooled and subsequently mixed with 50 μL of glacial acetic acid. Subsequently, the organic phase in the separatory funnel was collected and evaporated to dryness in a water bath at 40 °C under reduced pressure using a vacuum rotary evaporator. The resulting residue was immediately dissolved in 5 mL of ethyl acetate and subsequently dried using a nitrogen blower. The pellet was then suspended in 1 mL of methanol. The samples were filtered through a 0.22-μm filter (WondaDisc NY organic filter, SHIMADZU-GL Sciences, Shanghai, China) and prepared for HPLC analysis. Three replicates for each treatment were prepared, with each replicate being analyzed in triplicate.

### 2.6. Determination of PAT by Using HPLC

The analysis of patulin was performed utilizing a Shimadzu LC-20A HPLC system equipped with a ZORBAX SB-C18 column, following the methodology outlined by Zheng et al. (2020). The mobile phase comprised deionized water and acetonitrile in a 9:1 (*v*/*v*) ratio, with a flow rate set at 1 mL/min. Detection of patulin content in 20 μL of the sample was achieved using a PDA detector operating at a UV wavelength of 276 nm [34].

### 2.7. Fungal Isolation and Purification

Fungal isolation and purification were conducted following the methodology outlined by Deng et al. (2021), with minor modifications. Specifically, 20 g of homogenized samples was thoroughly mixed with 230 mL of sterile distilled water. Subsequently, 10 mL of the mixture was subjected to ten-fold serial dilutions. From these dilutions, 1 mL was spread onto PDA medium supplemented with 40 μg/mL chloramphenicol to inhibit the bacterial growth. The samples were then incubated at 25 °C for 5 days. Fungal colonies exhibiting distinct morphologies were subsequently subcultured onto fresh media to obtain pure isolates. After a four-day incubation period, various fungal species were differentiated based on their colony morphology, color, size, and texture. Pure isolates were subsequently numbered and preserved at −20 °C for further identification [35].

### 2.8. Identification of Fungi by Morphological Observation

Fungal spores were collected using a sterilized inoculating needle and subsequently immersed in sterilized distilled water. The spores were then fully oscillated to create a spore suspension. The spores were then cultured on PDA fresh medium, and pure isolates were acquired after 5 days of incubation at 25 °C. After culture, various strains were identified based on their colony morphology, color, size, and texture, and these features were recorded using smartphone photography. For microscopic analysis (BM2000, Nanjing Jiangnan Yongxin Optical Co., Ltd., Nanjing, China), segments of the medium with robust mycelial growth were chosen, and small pieces were cut using a scalpel on a clean slide. The microscopic morphology was observed and photographed for record-keeping.

### 2.9. Identification of Fungi by Using ITS Sequence

Molecular identification was performed based on the nucleotide sequence of the internal transcribed spacer (ITS) region. Fungal DNA was extracted using the Rapid Fungi Genomic DNA Isolation Kit (Sangon Biotech Co., Ltd., Shanghai, China). The ribosomal rRNA gene was amplified utilizing universal fungal primers: internal transcribed spacer 1 (ITS1: TCCGTA GGTGAACCTGCGG) and internal transcribed spacer 4 (ITS4: TCCTCCGCTTATTGATATGC). The PCR reaction mixture comprised 12.5 μL of 2× Taq Mix buffer, 1.0 μL of each primer (5 μmol), 1.0 μL of genomic DNA template, and ddH2O to a final volume of 25 μL. PCR amplification was conducted utilizing a thermal cycler (ABI9700, Applied Biosystems, Co., Ltd., Foster City, CA, USA) under the following cycling conditions: an initial denaturation at 95 °C for 15 s, followed by 30 cycles consisting of denaturation at 95 °C for 15 s, annealing at 57 °C for 15 s, and extension at 72 °C for 1 min, with a final extension step at 72 °C for 5 min. To assess the quality of the amplicons, 5 μL of the PCR products was subjected to electrophoresis on a 1% standard agarose gel, employing a DNA ladder for size reference. The gels were electrophoresed at 100 V for 30 min and subsequently visualized using a UV transilluminator in conjunction with the Gel Smart system. Amplification products were submitted to Sangon Biotech (Shanghai) Co., Ltd. for sequencing. The resulting nucleotide sequences were subsequently compared against the ITS region of fungal type and reference materials using the nucleotide BLAST program available at the National Center for Biotechnology Information (NCBI) database (http://www.ncbi.nlm.nih.gov/BLAST, accessed on 1 March 2024). All of the ITS sequences of the isolated fungal were subjected to maximum likelihood analysis to alignment using the phylogenetic analysis software MEGA version 7.0, following the specified methodology. Sequences were aligned utilizing the MUSCLE software (version 3.8.31) as described by Edgar (2004) [36]. The subsequent maximum likelihood (ML) phylogenetic tree was inferred using the IQ-TREE software (version 1.6.10) with the following parameters: best-fit model TIM3e + I + G4, 5000 ultrafast bootstrap replicates, and 1000 SH-like approximate likelihood ratio tests (Nguyen et al., 2015) [37].

### 2.10. Statistical Analysis

All the extraction procedures were performed in triplicate, *p* < 0.05 was considered to be statistically significant. Data are expressed as the mean ± standard deviation (shown as error bars in plots).

## 3. Results

### 3.1. OTA Contamination in the Cereal, Nut, Coffee, and Wine Food Samples

Table 1 shows OTA levels in 99 samples, detecting contamination in nine types of cereals and derivatives. These include two corn residue samples, one corn flakes sample, one millet sample, one black rice sample, one brown rice sample, and three oat samples, with OTA levels between 1.22 and 6.4 µg/kg. Only the black rice sample exceeded the regulatory limit of 5 µg/kg. OTA contamination was found in eight out of 23 types of nuts: one pistachio, two walnuts, three macadamia nuts, one pecan, and one melon seed sample, with levels ranging from 1.55 to 12.04 µg/kg. Three samples (one walnut and two macadamia nuts) exceeded the permissible limit of 5 µg/kg. OTA contamination was found in six of sixteen coffee samples, with concentrations between 4.13 and 48.17 µg/kg. Five of these exceeded the 5 µg/kg regulatory limit. All three wine samples also showed OTA contamination, with levels above the 2 µg/kg limit.

### 3.2. PAT Contamination in the Fruits and Fruit Derivate Foods

Table 1 details PAT presence in 50 food samples. PAT was found in 1 of 10 apple fruits at 192.78 µg/kg and in 6 of 24 apple-derived products (5 apple juices and 1 apple cider) with concentrations between 26.85 and 140.44 µg/kg. Four samples, including three apple juices and one apple cider, had PAT levels above the 50 µg/kg regulatory limit. Among 13 hawthorn derivative samples, three were contaminated with PAT, ranging from 29.83 to 88.56 µg/kg. Overall, 10 samples contained PAT, with seven exceeding the 50 µg/kg limit. PAT contamination was found in 16.67% of samples, with 11.67% exceeding the PAT limit.

### 3.3. Fungal in the Cereal, Nut, Coffee and Wine Food Samples

**Figure 2:**
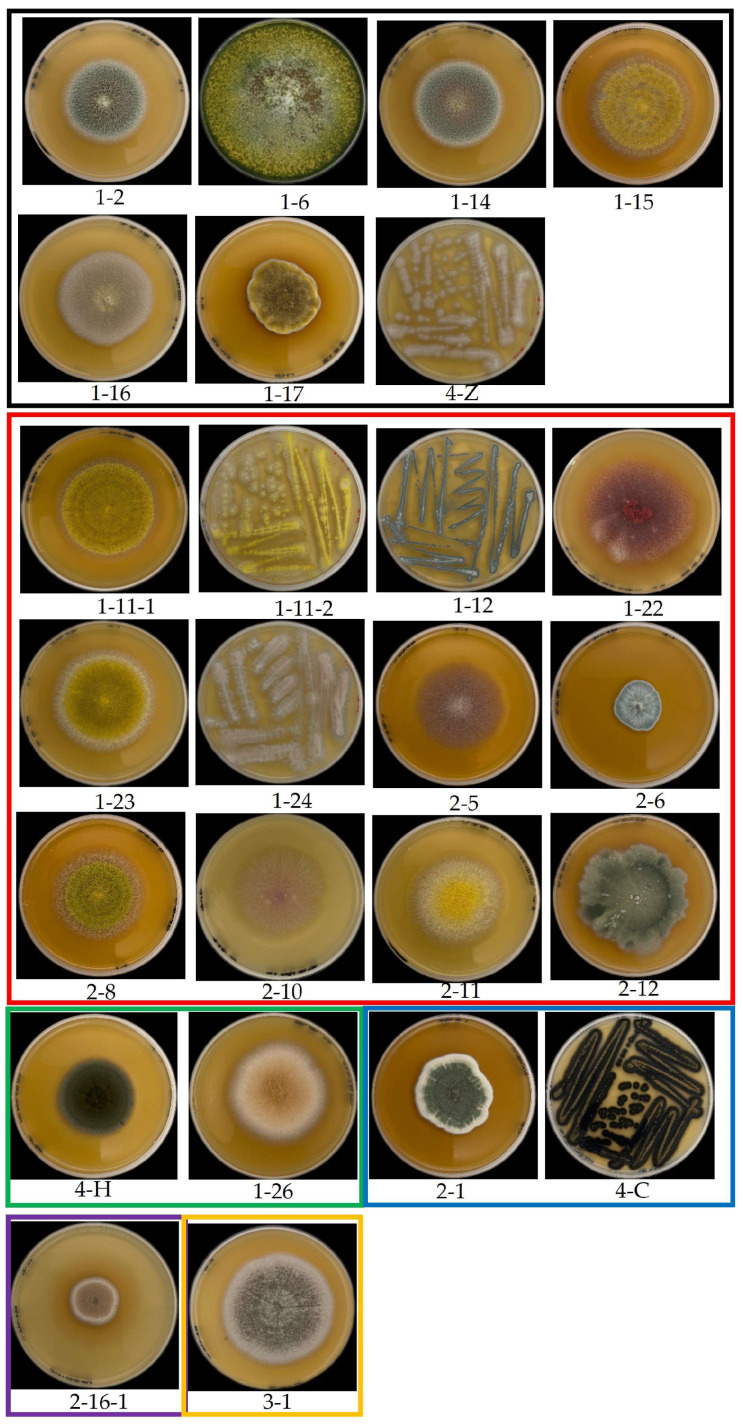
Fungal isolated from the cereal or nut food samples. The samples in the black, red, green, blue, purple and orange box represent the fungal isolated from the corn grit, corn flour, black rice, sticky rice, wheat flour, and pistachio nuts, respectively.

**Figure 3:**
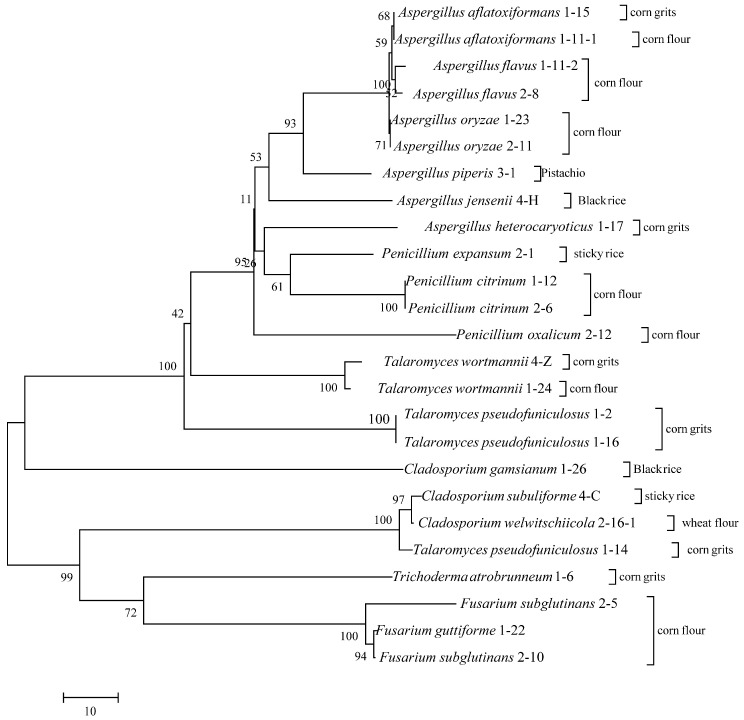
Evolutionary relationships of fungal isolated from the cereal or nut food samples.

Twenty-eight fungal isolates exhibiting diverse colony morphologies were obtained from the samples. Specifically, 8, 12, 2, 2, 1, and 1 isolate with distinct colony morphologies were isolated from corn grit, corn flour, black rice, sticky rice, wheat flour, and pistachio nuts, respectively. The colony morphologies of all isolated strains are detailed in Figure 2, and the evolutionary tree results of all strains are shown in Figure 3. The predominant genera in cereals included *Talaromyces*, *Fusarium*, *Aspergillus*, and *Penicillium*. Among them were nine strains of *Aspergillus* and four strains of *Penicillium*, which are also common fungi found in cereals. OTA is mainly a secondary metabolite produced by *Aspergillus* and *Penicillium* and is biosynthesized by primary metabolites formed in the proliferation process of mold as precursor substances. In addition, grain foods are also contaminated by *Talaromyces* and *Fusarium*. The colonies of strains 1-2, 1-14, and 1-16 exhibited neat edges and featured green or pink floccular, which were identified as *Talaromyces pseudofuniculosus*. Strains 1-11-2 and 2-8 were identified as *Aspergillus flavus*, characterized by yellow-green, fluffy-like colonies with neat edges, devoid of soluble pigments or exudates. Strain 1-6 was identified as *Trichoderma atrobrunneum*, which exhibited yellow mycelia. Strains 1-15 and 1-11-1 exhibited similar colony, mycelium morphology to strains 1-11-2 and 2-8; however, they have been identified as *Aspergillus aflatoxiformans*. Strains 1-23 and 2-11 have been classified as *Aspergillus oryzae*, characterized by yellow colonies with a fluffy texture and clean edges but lacking furrows, soluble pigments, and exudates. The colony of strain 1-17 displayed no furrows and had irregular edges, with a black-yellow coloration and a velvety texture, but it also lacked soluble pigments and exudates, which identified it as *Aspergillus heterocaryoticus*. Strains 4-Z and 1-24 were identified as *Talaromyces wortmannii*, characterized by colonies exhibiting pink and blue pigmentation, irregular edges, and a velvety texture. Colonies of strains 2-5 and 2-10 were powdery white, fluffy, with irregular edges, and lacked soluble pigments and exudates and were identified as *Fusarium subglutinans*. Strain 1-22 was identified as *Fusarium guttiforme*, exhibiting a colony and mycelium morphology similar to strains 2-5 and 2-10. Strain 4-H was identified as *Aspergillus jensenii*, characterized by a lighter colony color from the center outward and a neat colony edge. Strain 1-26 was characterized as *Cladosporium welwitschiicola*, with a brown, round, velvet-like colony, high mycelium density, and neat edges, but lacking pigment and exudate. Strain 4-C was identified as *Cladosporium montecillanum*, characterized by a black colony with neat edges and pleomorphic spores arranged axially along the mycelium. Strain 2-16-1 was identified as *Cladosporium gamsianum*, which exhibited a brown colony with neatly defined edges and dendritic mycelia, devoid of soluble pigments and exudates. Strains 1-12 and 2-6 were identified as *Penicillium citrinum*, featuring radiating furrows and a bluish-white colony with neat edges. Strains 2-12 were identified as *Penicillium oxalicum*, characterized by colonies exhibiting a grayish-green hue with uneven color distribution and fluffy, irregular edges. Strain 2-1 was identified as *Penicillium expansum*, which formed cyan-colored colonies with irregular edges. Strain 3-1 was identified as *Aspergillus piperis*, which displayed colonies with regular edges, a brown coloration, radiate mycelia on the surface, and well-developed mycelia.

### 3.4. Fungal Diversity in Apples, Hawthorn, Pears, and Their Derivatives

**Figure 4:**
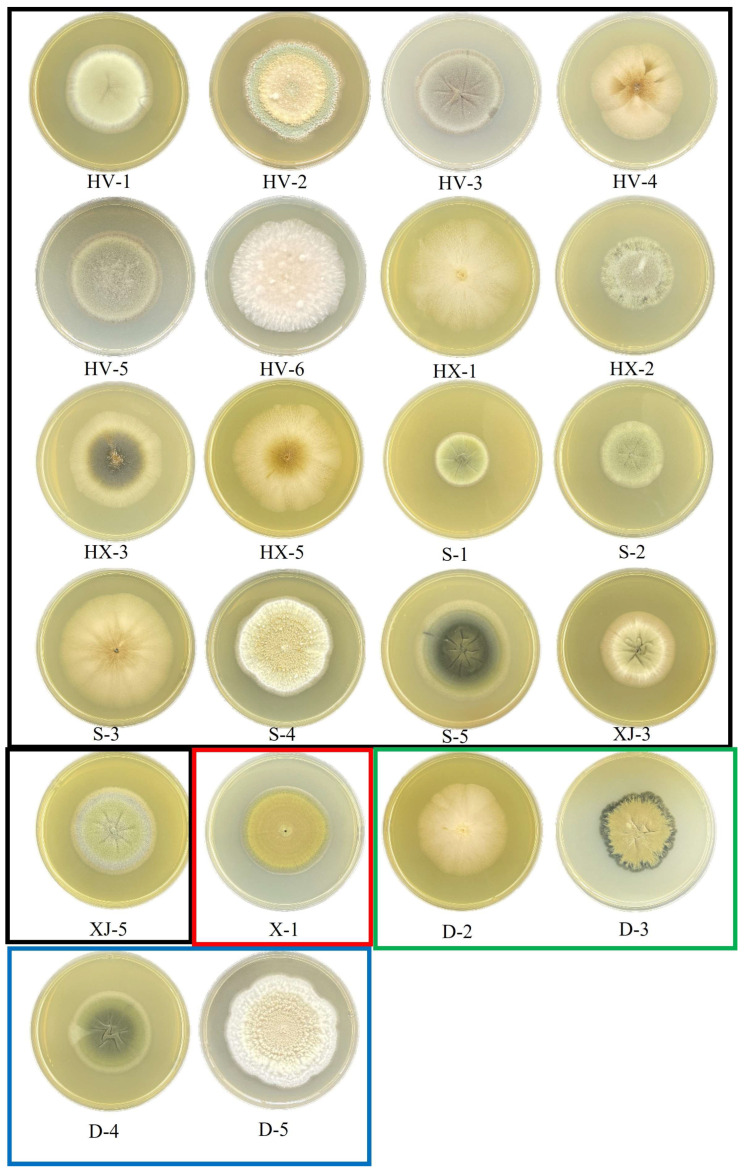
Fungal isolated from the fruit samples. The samples in the black, red, green, and blue box represent the fungal isolated from the apple, fresh-cut fruits and pear, respectively.

**Figure 5:**
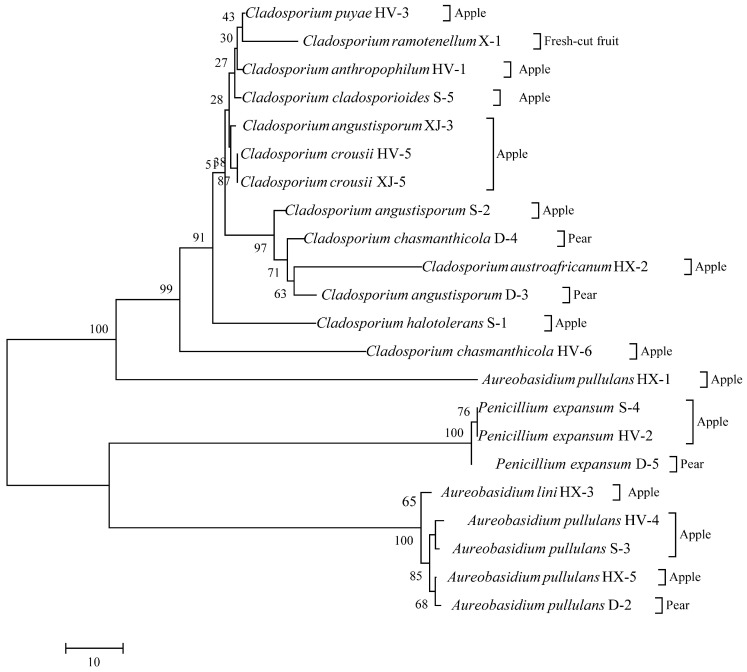
Evolutionary relationships of fungal isolated from the fruit samples.

Twenty-two fungal isolates exhibiting diverse colony morphologies were obtained from the fruit samples. Specifically, 17 isolates were derived from apple fruit, 4 from pear fruit, and 1 from fresh-cut apple. The morphological characteristics of the colonies, all isolated strains, are detailed in Figure 4; the evolutionary tree results of all strains are shown in Figure 5. The strain HV-1 was identified as *Cladosporium anthropophilum*, characterized by a flat, gray-velvet colony with central folds and neatly rounded edges. The mycelium of strain HV-1 exhibits branching, with wheat-like granular spores developing laterally along the mycelium. Strains HV-2, S-4, and D-5 have been identified as *Penicillium expansum*, characterized by colonies that are yellowish-white and velvety, with radial wrinkles on the surface. Strain HV-3 has been identified as *Cladosporium puyae*, which forms flat, gray velvet colonies with central folds and neatly rounded edges. The mycelium of HV-3 is segmented by septa. The strains HV-4, HX-1, HX-5, S-3, and D-2 were identified as *Aureobasidium pullulans*. These strains exhibit low colony density, with colonies appearing white and fluffy, and darkening at the center during culture. Strains HV-5 and XJ-5 were identified as *Cladosporium crousii*, characterized by gray, velvety colonies that are round with regular edges. Strain HV-6 was identified as *Cladosporium gossypiicola*, which forms white, fluffy colonies with protruding surfaces and irregular edges. Strains HX-2, S-2, XJ-3 and D-3 were identified as *Cladosporium angustisporum*, characterized by a gray velvet colony with neatly rounded edges. Strain HX-3 was identified as *Aureobasidium lini*, which forms a colony with low density, a white fluffy appearance, and a darker center. Strain S-1 was identified as *Cladosporium halotolerans*, which forms a light-yellow velvet colony with radial folds in the center and neatly rounded edges. Strain S-5 was identified as *Cladosporium cladosporioides*, characterized by a gray velvet colony with radial folds at the center and neatly rounded edges. Strain X-1 was identified as *Cladosporium ramotenellum*, which exhibits a yellow velvet colony with a flat surface and neatly rounded edges. D-4 has been identified as *Cladosporium chasmanthicola*. This colony presents a gray, velvety texture with radial folds in the central area and neatly rounded edges.

## 4. Discussion

The production conditions of mycotoxins include the biological factors of raw materials, environmental factors in the planting and harvesting process, production technology and storage conditions, as well as operations in the process of sales and use, etc. When the food is mildewed due to these factors, mycotoxins may be produced. OTA is a highly toxic mycotoxin that poses a significant threat to human health. Given its severe toxicity and pervasive contamination, substantial efforts have been undertaken to assess human exposure to this compound. Cereals such as rice, wheat, corn, rye, barley, oats, and millet, along with their derived products, constitute staple foods in human diets globally. Due to climatic conditions and storage practices, these cereals are particularly susceptible to fungal growth and subsequent OTA contamination, rendering them primary sources of human exposure [12]. A comprehensive review of OTA contamination in cereals across 100 countries, including the USA, Canada, Iran, Algeria, Poland, and China, was conducted. Notably, China holds the highest rice production yield globally [11]. An analysis of 370 rice samples from six provinces in China revealed that 4.9% of the samples were contaminated with OTA, with only one sample exceeding the European Commission’s maximum residue limit (MRL) [38]. However, OTA was not detected in staple food items such as rice, corn flour, glutinous rice, and rice flour in Yangzhou. OTA contamination is also present in various cereal products, including corn residue, corn flakes, millet, black rice, and brown rice. The average contamination level of OTA was found to be 0.2 μg/kg, with the highest observed level reaching 3.0 μg/kg. Notably, the OTA content did not exceed the maximum limit of 5 μg/kg as stipulated by Chinese food safety standards, except for a single black rice sample that exhibited an excessive OTA content of 6.4 μg/kg. In the examined cereal foods, the OTA positive rate was 9 out of 57 samples, while the rate of samples exceeding the OTA limit was 1 out of 57.

Following cereals, wine, coffee, and nuts constitute significant sources of OTA in the overall dietary intake [11]. The raw materials used in the production of wine, coffee, and nuts are particularly prone to infection by ochratoxigenic fungi, and the high sugar content in these matrices provides an ideal environment for OTA production. Contamination is likely to occur at the vineyard stage, and various factors, including geographical and climatic conditions, have been reported to influence OTA levels in wine, coffee, and nuts. Analysis of 223 wine samples from seven provinces revealed OTA concentrations ranging from 0.01 to 0.98 μg/L, with an average concentration of 0.15 μg/L [39]. Specifically, wine from Jilin province exhibited the lowest mean value due to its high latitude and low temperature. Another survey encompassed four provinces in China, revealing an OTA detection rate slightly higher than previously reported: 66% for red wine and 55% for white wine [40]. The average OTA content was 0.61 μg/L. Additionally, a separate survey collected 42 samples from the Hexi Corridor Region of China [41], demonstrating good quality with a maximum OTA concentration of 1.27 μg/L. In the current study, all three wine samples were contaminated with OTA, with concentrations ranging from 3.12 to 4.59 μg/L. Green coffee samples were collected from nine countries during 2015 and 2016 for the analysis of 31 mycotoxins. The countries included Vietnam (*n* = 24), Brazil (*n* = 19), Colombia (*n* = 7), Ethiopia (*n* = 5), Ivory Coast (*n* = 4), China (*n* = 4), Indonesia (*n* = 4), Mexico (*n* = 2), and Guatemala (*n* = 2) [42]. Liquid chromatography-mass spectrometry (LC-MS) results indicated that OTA was present in 28% of the samples, with an average concentration of 1.3 g/kg. In the current study, 6 out of 16 coffee samples were contaminated with OTA, exhibiting concentrations ranging from 1.13 to 4.17 μg/L. It was reported that 1.6% of 253 samples of nuts and dried fruits in China were contaminated with OTA [43]. The maximum OTA concentration observed was 9.39 µg/kg, with a mean concentration of 6.23 µg/kg. In the current study, 8 out of 23 nut samples were found to be contaminated with OTA. Specifically, OTA concentrations in 2 walnut samples and 3 macadamia nut samples exceeded the limits set by Chinese standards. In this study, the coffee samples tested contained a high OTA content, which may be generated during the growing, harvesting, storage or processing of coffee beans, and the content of OTA could not be completely reduced by roasting coffee beans.

Mahato et al. (2021) provided a comprehensive summary of the global dissemination of PAT, highlighting significant levels of PAT contamination in fruits and their derivatives across both developed nations, including the United States, Canada, Portugal, and Belgium, as well as developing countries such as South Africa, Argentina, and Turkey. Studies have indicated that PAT is predominantly ingested through apples and apple-derived products [16]. Consequently, the issue of PAT contamination has garnered substantial international attention. In this study, 60 samples, including apple and hawthorn products [30], were analyzed. PAT was detected in 10 samples, with 7 samples exceeding the permissible limits. The highest concentration of PAT, measured at 192.78 µg/kg, was found in the fresh cut samples. The remaining healthy portion of a decayed apple, once the rotten section is excised, may be utilized for producing fresh-cut apples. However, it has been observed that significant levels of mycotoxins can still be present in this ostensibly healthy portion. Furthermore, an analysis of apple juice samples revealed that five out of thirteen were contaminated with PAT, with three samples exceeding the permissible limits. These findings underscore the necessity for vigilant monitoring of PAT contamination in both freshly cut apples and apple juice.

The isolation and identification of mold in food can offer scientific evidence for the assessment and monitoring of mold and mycotoxin contamination in food products. In this study, mycoides were isolated and identified in 145 samples across all 31 categories, but were found in only 9 specific sample categories: corn flour, corn residue, black rice, glutinous rice, flour, pistachio, apple, fresh-cut fruit, and pear. No mold contamination was detected in the remaining samples. Additionally, mycotoxin residues were found in 20 out of the 31 sample categories. The contamination of mycotoxins in food exhibits characteristics associated with multiple stages, including the harvesting, processing, and storage of food raw materials. In this study, mycotoxins were detected in several samples; however, no corresponding toxigenic mold was isolated. This finding suggests that the source of mycotoxins in these samples is likely attributable to the raw materials themselves, rather than to mycotoxins produced during the storage process as a result of mold contamination. Among the identified molds, *Aspergillus aflatoxiformans*, *Aspergillus intermedius*, *Aspergillus flavus*, *Aspergillus oryzae*, *Aspergillus jensenii*, and *Aspergillus piperis*, among others, have been reported to produce aflatoxin [18]. These findings suggest that food contaminated with the aforementioned molds may also be contaminated with aflatoxin.

Seventeen strains of mold were isolated from the surface of apples, two from fresh-cut fruits, and four from the surface of pears. The fungi isolated from the surfaces of apples and pears were identified as belonging to the genera *Cladosporium*, *Penicillium*, *Aureobasidium*, *Alternaria*, *Curvularia*. Notably, *Penicillium expansum*, a toxigenic strain known for producing PAT, was isolated. Numerous studies have documented that *P. expansum* can produce PAT. Under favorable environmental conditions, this fungal can infect apples through mechanical wounds and subsequently produce toxins. Therefore, preventing mold spoilage and toxin production on the surface of apples is crucial. Although no mold was isolated from apple juice and apple cider vinegar, the presence of PAT contamination in both products suggests that the primary source of PAT contamination originated from the raw materials. One *P. expansum* strain was isolated from fresh-cut fruit, and a significant level of PAT contamination was detected in these fresh-cut fruits. This observation leads us to hypothesize that the use of ostensibly healthy fruits, from which only the visibly rotten parts were removed, may contribute to the contamination. It is likely that the healthy portions of these fruits were already infected with substantial quantities of mycelium even after the removal of the visibly decayed sections. Upon significant accumulation of PAT in the decayed regions, it subsequently disseminates into the adjacent healthy tissues [44]. Consequently, even the ostensibly healthy tissue excised from the decaying sections retains substantial levels of toxins. The inadvertent contamination of freshly cut fruits with PAT is predominantly attributable to the negligence or insufficient food safety knowledge among producers, who erroneously believe that excising the decayed portions renders the remaining tissue safe for consumption. This underscores the critical need for comprehensive food safety training for personnel involved in food production.

## 5. Conclusions

This study analyzed the mold and OTA contamination in cereal products, and the mold and PAT contamination in apple and hawthorn products in Yangzhou market. The study found that OTA and PAT contamination existed in food in this city. Corn processed products are the main source of OTA contamination, apple processed products are the main source of PAT contamination, and the toxin content in some samples exceeded the Chinese food safety limit standard. In addition, strains of *Fusarium*, *Aspergillus* and *Penicillium*, which have been isolated from the genus, may produce toxins during the food shelf life, threatening the health of consumers. The findings provide valuable insights for government market regulation and informed consumer decisions. Furthermore, these findings indicate that OTA and PAT toxins are one of the important risk factors that threaten consumer health. The key to avoid further harmful effects is to strengthen the sampling inspection of key contamination sources and remove the food products with excessive toxin in time. In addition, products contaminated with toxins should be traced to seek to solve the situation of toxin contamination at the source, including control of the contamination of toxin-producing fungi.

## Figures and Tables

**Table 1:** OTA or PAT content in the food samples.

Sample Classification	Sample Name	Number of Sample Type	Mean ± SD (µg/kg)	OTA Content (µg/kg)	Number of Positive	Number of Exceedance	Risk
Cereals related foods	Rice	7	/	/	/	/	/
	Corn grit	6	3.22 ^b^ ± 0.63	2.90–3.53	2	/	/
	Corn flour	7	/	/	/	/	/
	Corn flakes	3	1.32 ^c^ ± 0.07	1.32	1	/	/
	Sticky rice	3	/	/	/	/	/
	Millet	7	2.04 ^c^ ± 0.05	2.04	1	/	/
	Black rice	5	6.40 ^a^ ± 0.33	6.40	1	1	serious
	Brown rice	5	1.70 ^c^ ± 0.22	1.70	1	/	/
	Wheat flour	5	/	/	/	/	/
	Oat	9	2.26 ^c^ ± 1.72	1.22–4.25	3	/	/
Total		57	2.80 ± 1.91	1.22–6.40	9	1	/
Nuts	Pistachio nuts	3	1.55 ^c^ ± 0.06	1.55	1	/	/
	Sinian wood	2	/	/	/	/	/
	Walnut	2	8.17 ^a^ ± 6.24	3.75–12.58	2	1	serious
	Broad bean	2	/	/	/	/	/
	Macadamia nut	5	8.19 ^a^ ± 3.70	4.66–12.04	3	2	serious
	Cashew nut	4	/	/	/	/	/
	Pecan fruit	3	3.35 ^b^ ± 0.13	3.35	1	/	/
	Melon seed	2	4.20 ^b^ ± 0.11	4.20	1	/	/
Total		23	4.41 ± 3.40	1.55–12.58	8	3	/
Coffees	Hazelnut coffee	4	35.61 ^a^ ± 0.21	35.61	1	1	serious
	Latte	4	40.01 ^a^ ± 0.27	40.01	1	1	serious
	White coffee	3	26.15 ^b^ ± 31.14	4.13–48.17	2	1	serious
	White peach coffee	5	20.72 ^c^ ± 17.35	8.45–32.99	2	2	serious
Total		16	20.20 ± 18.12	4.13–48.17	6	5	/
Wine	Wine	3	4.02 ^a^ ± 0.79	3.12–4.59	3	3	serious
Total		3	4.02 ± 0.79	3.12–4.59	3	3	/
Sample classification	Sample Name	Number of Sample Type	Mean ± SD (µg/kg)	PAT content (µg/kg)	Number of positive	Number of exceedance	risk
Fruits	Red Fuji Apple	5	/	/	/	/	/
	Fresh-cut apple	5	192.78 ± 7.30	192.78	1	1	serious
	Pear	3	/	/	/	/	/
Total		13	192.78 ± 7.30	192.78	1	1	/
Apple products	Apple juice	13	84.16 ± 52.69	26.85–140.44	5	3	serious
	Cider	3	87.14 ± 3.03	87.14	1	1	serious
	Apple jelly	3	/	/	/	/	/
	Apple cider vinegar	5	/	/	/	/	/
Total		24	85.28 ± 39.90	26.85–140.44	6	4	/
Hawthorn products	Hawthorn preserves	13	63.03 ± 30.11	29.83–88.56	3	2	serious
Total		13	63.03 ± 30.11	29.83–88.56	3	2	/

Lowercase letters (^a^, ^b^, ^c^) represents significant differences in toxin content among the positive samples (*p* < 0.05). The term “serious” refers to samples with toxin levels exceeding China’s safety standards.

## Data Availability

The original contributions presented in the study are included in the article, further inquiries can be directed to the corresponding author.
